# Resveratrol Reduces ROS by Increasing GSH in Vitrified Sheep Embryos

**DOI:** 10.3390/ani13233602

**Published:** 2023-11-21

**Authors:** Andrea Carolina González-Garzón, Julio Porfirio Ramón-Ugalde, Demetrio Alonso Ambríz-García, José Roberto Vazquez-Avendaño, José Ernesto Hernández-Pichardo, José Luis Rodríguez-Suastegui, César Cortez-Romero, María del Carmen Navarro-Maldonado

**Affiliations:** 1 Division of Biological and Health Sciences, Universidad Autónoma Metropolitana Unidad Iztapalapa, Mexico City 09310, Mexico; andrea.gonzalez.garzon@unillanos.edu.co; 2 Division of Posgraduate Studies and Research, Tecnológico Nacional de México Campus Conkal, Yucatán 97345, Mexico; julio.ramon9@gmail.com; 3 Department of Biology of Reproduction, Universidad Autónoma Metropolitana Unidad Iztapalapa, México City 09310, Mexico; deme@xanum.uam.mx (D.A.A.-G.); robertmizer@gmail.com (J.R.V.-A.); 4 Department of Agriculture and Animal Production, Universidad Autónoma Metropolitana Unidad Xochimilco, México City 04960, Mexico; mvzjehp@yahoo.com (J.E.H.-P.); embrioninvitro@hotmail.com (J.L.R.-S.); 5 Campus San Luis Potosí, Colegio de Postgraduados, Salinas de Hidalgo 78622, Mexico; ccortez@colpos.mx

**Keywords:** oxidants, antioxidants, enzymes, in vitro, embryo vitrification

## Abstract

**Simple Summary:**

The in vitro production of mammalian embryos generates oxidative molecules known as reactive oxygen species that damage the cells developed in in vitro cultures because of the culture system itself and other biotechnologies commonly used for animal- assisted reproduction, such as cryopreservation of embryos by vitrification. That is why antioxidants must be added to the in vitro system. Thus, we evaluated if the addition of a particular antioxidant (resveratrol) to the oocytes when they were matured in vitro and then used for producing embryos by in vitro fertilization and vitrification could protect the embryos from the damage caused by vitrification. The results showed that resveratrol, when added to the culture media of oocytes matured in vitro, protects embryos produced by in vitro fertilization from damage caused by vitrification by decreasing reactive oxygen species levels and facilitating the generation of the embryo antioxidant enzymes like glutathione. This means that resveratrol can be used to help embryos overcome vitrification damage.

**Abstract:**

The in vitro production and cryopreservation of mammalian embryos generates reactive oxygen species (ROS) due to conditions of the system that can overcome their antioxidant protection. Resveratrol is an antioxidant used in in vitro systems to improve blastocyst rates, but its effect on antioxidant enzymes such as glutathione (GSH) in embryos produced by in vitro fertilization (IVF) after vitrification has not been reported. The objective of this study was to evaluate the effects of resveratrol on the in vitro maturation medium (IVM) of sheep oocytes (*Ovis aries*) on the levels of ROS and GSH in embryos produced by IVF subjected to vitrification. Resveratrol was added at 0 µM, 0.25 µM, 0.5 µM, and 1 µM during oocyte in vitro maturation (IVM). Matured oocytes were fertilized with thawed ram sperm. Embryos were cultured in sequential media until blastocysts, were then vitrified for 24 h, and, after heating, they were stained with DCFH-DA (2′,7′-dichlorodihydrofluorescein diacetate) to determine the presence of ROS and with Cell Tracker Blue^®^ for the presence of GSH. The quantitative values of ROS and GSH were obtained through the Image J image processor. The results showed that resveratrol increased GSH and decreased ROS production (*p* < 0.05) in a dose-dependent manner. It is concluded that its use in sheep oocytes during IVM has a beneficial effect on embryos produced by IVF subjected to vitrification by decreasing reactive oxygen species levels and facilitating the generation of embryo antioxidant enzymes like glutathione.

## 1. Introduction

The in vitro production of embryos is a biotechnology that impacts livestock systems by promoting genetic improvement. The cryopreservation of gametes and embryos has been widely developed worldwide in recent years [1]; however, intrinsic processes can cause damage to cells and reduce embryo viability. One of the main factors detected in sheep is the increase in reactive oxygen species (ROS) [2], derived from an imbalance with endogenous embryonic antioxidants.

To reduce the damage, antioxidants such as resveratrol have been added to the in vitro culture media for in vitro maturation of sheep oocytes. Martínez et al. [3] observed that adding resveratrol at 0.25 and 0.5 μM to sheep oocytes (*Ovis aries*) during in vitro maturation (IVM) improved their quality and promoted the compaction of morulae produced by somatic cell nuclear transfer (TNCS). Likewise, it is known that the supplementation of resveratrol (0.25 and 0.5 μM) in the IVM medium of mature cow and goat oocytes reduces intracellular ROS levels, increases glutathione (GSH) concentrations, and stimulates embryonic development and gene expression [4]. In sheep, Zabihi et al. [5] did not find differences in the IVM rate but found significant differences in the blastocyst rate at concentrations of 0.25 and 0.5 μM of resveratrol, the quality of these being better at 0.5 μM.

In pigs, it is known that the addition of resveratrol during IVM and/or vitrification modulates the apoptotic process, improving the cryotolerance of vitrified porcine oocytes, while in bovine oocytes, it reduces ROS levels and increases blastocyst rates and the number of embryonic cells. Particularly for bovine embryos, supplementation with low concentrations of resveratrol in the medium for in vitro development reduces ROS production and increases beta-oxidation levels, leading to higher survival rates after vitrification [6]. In the sow, Kwak et al. [7] used resveratrol at 0.1, 0.5, and 2.0 μM during IVM of oocytes to evaluate its effect on ROS and GSH levels and gene expression in mature oocytes, cumulus cells, and blastocysts derived from the IVF. They observed that 2.0 μM of resveratrol improved the developmental potential of embryos, decreased ROS, and increased GSH, but the authors did not prove its effect on vitrified embryos.

Abdul Rahman et al. [8] say that “glutathione constitutes the major non-protein sulphydryl compound in mammalian cells, which confers protection against oxidative damage”. Working with mice, authors directly added 0.01 mM of exogenous L-glutathione enzyme to the in vitro development culture media, showing that these improved intracellular GSH contents and vitrification outcomes in preimplantation embryos, as observed through embryo morphology and preimplantation development. They also observed that this supplementation reduced the ROS levels in these embryos.

García-Martínez et al. [9] proved that glutathione ethyl ester supplemented to the IVM media protects bovine oocytes against oxidative stress induced by subsequent vitrification/warming by preserving the mitochondrial distribution pattern, diminishing the cytoplasmic and mitochondrial ROS contents, and making embryonic developments like non-vitrified oocytes.

Other antioxidants have been proven in porcine experiments, where Xiang et al. [10] found that when adding 1.5 µM of astaxanthin to the culture medium the developmental competence of parthenogenetic zygotes improved (51.7% blastocysts). This concentration also improved the blastocyst formation rate of vitrified cloned zygotes (23.3% blastocysts).

In a bovine study, Giraldo [11] determined the damage in vitrified embryos due to malondialdehyde (MDA) produced during the oxidation of phospholipids. By measuring MDA concentrations in media subjected to vitrification and post-vitrification with and without embryos, he found that, in the first case, it produced 35.26% more MDA compared to the second. He also observed that, at a higher concentration of cryoprotectants (dimethyl sulfoxide (DMSO) + DMF-dimethylformin, 20% each), the concentrations of MDA, ROS, and thiobarbituric acid (TBAR) decreased.

Given that resveratrol shows an antioxidant effect that favors the IVM of oocytes as well as the quality of embryos produced from them, in this study, we evaluated the effects of resveratrol on the in vitro maturation medium (IVM) of sheep oocytes (*Ovies aries*) on the levels of ROS and GSH in embryos produced by IVF subjected to vitrification. *O. aries* is an animal species that our research group has worked with for a long time, and this effect has not been described in devitrified IVF embryos.

## 2. Materials and Methods

### 2.1. Oocyte Collection

Ovaries were collected from adult domestic sheep (*Ovis aries*) slaughtered at a local slaughterhouse and transported to the laboratory within 2 h at 30–35 °C in saline solution (0.9% NaCl) with 1% antibiotics and antifungal (10,000 IU/mL of penicillin, 10 mg/mL of streptomycin sulfate, and 25 μg/mL of amphotericin) (Antibac-Antifun 100×, In Vitro, SA, Mexico City, Mexico). Once in the laboratory, the ovaries were washed and the cumulus–oocyte complexes (COCs) were collected by puncturing the ovarian follicles (2 to 5 mm diameter) with a 10 mL syringe and a 20 G needle containing TCM-199^®^ with HEPES 25 mM (In Vitro SA, Mexico City, Mexico) with heparin (100 IU/mL) [3]. The COCs were selected based on the ASEBIR [12] criteria.

### 2.2. In Vitro Maturation (IVM) of Oocytes

In vitro maturation (IVM) was carried out in TCM-199^®^ (In Vitro SA, Mexico City, Mexico) supplemented with cysteine (0.57 mM), D-glucose (3.05 mM), polyvinyl alcohol (0.1%), sodium pyruvate (0.91 mM), 10% fetal bovine serum (FBS, Microlab SA de CV, Mexico City, Mexico), 10 ng/mL of epidermal growth factor (EGF), 0.1 IU/mL^−1^ of recombinant follicle-stimulating hormone (rFSH, Gonal-F Merck Serono, Darmstadt, Germany), 5 IU mL^−1^ of equine chorionic gonadotropin (eCG, Gonaforte Parfarm SA, Mexico City, Mexico), and antibiotics (0.6%) (Antibac-Antifun 100×, In Vitro, SA, México City, México). The IVM medium was added with the different concentrations of resveratrol (0.0, 0.25, 0.50, and 1.0 μM). This last concentration was chosen since, in a previous study [3], 2 μM of resveratrol had a negative effect on IVM and embryonic development. The COCs were distributed in groups of 25 to 30 in cells of four-cell boxes (Nunc, Thermo Fisher Scientific, Waltham, MA, USA) in 400 μL of IVM medium from each treatment covered with mineral oil, culturing them for 24 h at 38.5 °C, 5% CO_2_, and >70% humidity at saturation. After IVM, 10% of the COCs from each treatment were stripped from the cumulus cells (CCs) in 0.5 mg/mL^−1^ of hyaluronidase, to determine the rate of in vitro maturation (IVM—rate of oocytes in metaphase II, or MII) [3] (Martínez et al., 2019) by observing the expansion of the CCs and the presence of the first polar body [12]; the rest was used for IVF. There were 8 replicates made.

### 2.3. In Vitro Fertilization (IVF) and In Vitro Development (IVD) of Embryos

The COC showing expanded CC were washed in 50 µL of BO-IVF™ in vitro fertilization (IVF) medium (IVF Bioscience, Cornwall, United Kingdom), covered with mineral oil, and tempered at 38.5 °C. Then, 30 oocytes were distributed in four-cell culture dishes in 100 µL of the IVF medium [13]. Straws with semen from fertile rams were thawed at 37 °C before use. Post-thawing, sperm motility was evaluated. “Swim up” was performed in Eppendorf tubes (Sorenson Biosciences, Inc., Salt Lake City, UT, USA) [2] in the IVM medium recovered from the oocytes that were matured from the control group [14]. Ten µL were taken from the upper part of the tubes with the motile sperm fraction (0.5 × 106 mL^−1^ sperm) and added to the IVF medium containing the oocytes from each treatment, incubating for 24 h under the same conditions [2]. Fertilized oocytes (zygotes) were washed twice in 50 µL of cleavage medium (Cook Medical^®^, Bloomington, IN, USA) for the in vitro development (IVD) and transferred to a new culture dish by placing 25 zygotes per 50 µL of IVD medium covered with mineral oil. They were incubated for 4 days under the same conditions. On day 4 of culture, the embryos were transferred to another dish with 50 µL of blastocyst medium (Cook Medical^®^, Bloomington, IN, USA), and the IVD rate (early blastocysts) and embryonic morphology were evaluated in each treatment [2]. The embryos remained in blastocyst medium (Cook Medical^®^, Bloomington, IN, USA) for an additional 24 h. The next day (day 5 of culture), early blastocysts were prepared for vitrification. There were 8 replicates made.

### 2.4. Vitrification and Devitrification of Embryos

Following Bhat et al. [15], 10% of the best-quality early blastocysts of each experimental group were vitrified using the open pulled straw (OPS) technique [16], which consisted of washing the blastocysts in 800 µL of holding medium (TCM-199 without HEPES, supplemented with 10% FBS). They were then sequentially equilibrated, first in 600 µL of holding medium supplemented with 7.5% of ethylene glycol and 7.5% of DMSO for 3 min, then in 600 µL of holding medium with 0.342 g/mL of sucrose, 16.5% of ethylene glycol, and 16.5% of DMSO for 20 s. Immediately afterwards, 2 µL drops were formed with the embryos and collected by capillarity in an OPS. These were then placed inside straws (0.25 mL) that were heat-sealed and subsequently immersed in the liquid nitrogen tank for 24 h.

For devitrification, the OPS were thawed using the three-step technique [17]. First, each straw was removed from the nitrogen tank, the sealed end was cut, and the OPS was removed from the inside of the straw. The tip of the straw was introduced into 400 µL of holding medium supplemented with 50% sucrose so that the embryos could descend via gravity, after which they were left for 5 min. The embryos were then transferred to 1000 µL of holding medium supplemented with 25% sucrose for 5 min. Finally, the embryos were washed in 400 µL of holding medium without sucrose for 5 min. The devitrified early blastocysts were cultured again for 24 h in blastocyst medium (Cook Medical^®^, Bloomington, IN, USA) under the same conditions until they reached the late blastocyst (expanded and hatched blastocysts) stages [18]. There were 8 replicates made.

### 2.5. Quantification of ROS and GSH in Devitrified Embryos

A sample of 10% of the devitrified early blastocysts from each group was taken. Following the methodology described by Mukherjee et al. [4], at the end of post-vitrification IVD (day 7 of culture), 10 µM of DCFH-DA (2′,7′-dichlorodihydrofluorescein diacetate) and 10 µM of the Cell Tracker Blue^®^ (Invitrogen, Eugene, OR, USA) stain were added to the medium containing the embryos, and the culture dish was covered with aluminum foil and incubated for 30 minutes under the conditions described. Subsequently, the embryos were washed 3 times in 100 µL of DPBS (Dulbeco Phosphate Buffered Saline, In Vitro, SA, Mexico City, Mexico) and mounted on slides, which were covered with coverslips and sealed with nail polish. They were evaluated under an epifluorescence microscope with a 460 nm UV filter to evaluate ROS and 405 nm to evaluate GSH. Photographs were taken. The higher the fluorescence intensity, the more the presence of higher levels of ROS or GSH in the embryos was qualitatively considered. Fluorescence intensity was evaluated in an Image J image processor (version 1.53; Wayne Rasband, National Institute of Health, Kensington, MD, USA) to obtain semiquantitative data.

### 2.6. DAPI Staining of Nuclei

Prior to and after vitrification, following the methodology of Vazquez-Avendaño et al. [19] with some modifications, on day 7 of culture, the number of nuclei present in the blastocysts was determined. To accomplish this, embryos of each group were washed for 2 min in 100 µL of DPBS (Dulbeco Phosphate Buffered Saline In Vitro, SA, Mexico City, Mexico) and fixed for 24 h in 400 µL of 4% paraformaldehyde in the refrigerator. Subsequently, they were deposited on a slide in a 2 µL drop, removing the excess of the fixative. Ten µL of a 2.5% solution of 4′,6′-diamidino-2-phenylindole (DAPI) in DPBS were added to the slide and covered with a coverslip. A 405 nm UV filter was used to evaluate them. Once the images of each group were obtained, the nuclei of each embryo were counted.

### 2.7. Statistical Analysis

The response variables analyzed were the rate of in vitro maturation (IVM) of oocytes, embryonic segmentation, in vitro development (IVD), blastocysts with post-vitrification development capacity, presence of ROS and GSH, and embryonic quality. These variables were analyzed, considering the control group and the experimental groups (with the different concentrations of resveratrol), using Student’s *t*-test for paired samples. A one-way ANOVA was used, considering the intragroup response variables, followed by the Duncan test using the SAS Visual Analytical 2020.1 (SAS Institute Inc., Cary, NC, USA) program, at a significance of *p* < 0.05.

## 3. Results

### 3.1. Oocyte Collection

Eight hundred and fifteen ovaries were collected from adult domestic sheep (*O. aries*), with an average aspiration rate of 3 oocytes/ovary. The IVM rate of oocytes treated with different concentrations of resveratrol was analyzed; IVF was verified by the embryonic segmentation rate and the IVD rate.

### 3.2. In Vitro Maturation (IVM) of Oocytes, In Vitro Fertilization (IVF), and In Vitro Development (IVD) of Embryos

The IVM rate was similar in oocytes treated with 0.5 and 1 µM of resveratrol, and was higher compared to oocytes treated with 0 and 0.25 µM (*p* ≤ 0.05). The IVD, determined by the embryonic cleavage rate, was also similar for 0.5 and 1 µM of resveratrol and higher with respect to 0 and 0.25 µM (*p* ≤ 0.05). Regarding the blastocyst rate, although it did not show statistical differences between groups (*p* ≥ 0.05), it was observed that 0.25 µM of resveratrol reduced the rate of late blastocysts (Table 1).

### 3.3. Vitrification and Devitrification of Embryos

**Figure 1:**
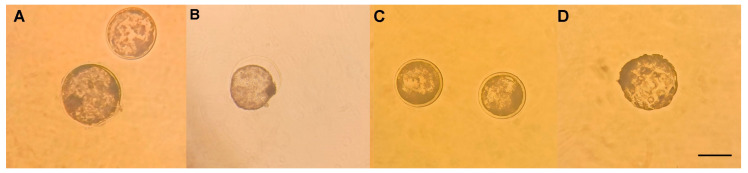
Late blastocysts produced by in vitro fertilization (IVF) from *Ovis aries* oocytes treated with resveratrol during IVM, after devitrification. (**A**) 0 µM resveratrol (late expanded blastocysts); (**B**) 0.25 µM resveratrol; (**C**) 0.5 µM resveratrol (expanded blastocysts); (**D**) 1 µM resveratrol (hatched blastocyst). Scale bar 100 µM.

No significant differences were found in the rate of late blastocysts after devitrification (*p* ≥ 0.05). However, at 1 µM of resveratrol it looks higher (Figure 1 and Table 2).

### 3.4. ROS and GSH Levels in Devitrified Embryos

**Figure 2:**
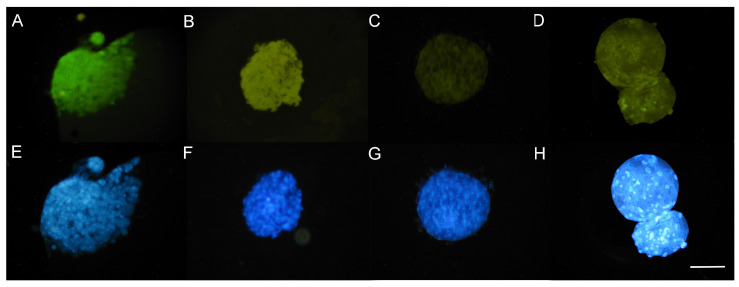
Qualitative evaluation of the presence of ROS and GSH in blastocysts produced by in vitro fertilization (IVF) from *Ovis aries* oocytes treated with resveratrol during in vitro maturation (IVM) before vitrification. (**A**): 0 µM; (**B**): 0.25 µM; (**C**): 0.5 µM; and (**D**): 1 µM resveratrol. (**A**–**D**): Green images DCFH-DA (2′,7′-dichlorodihydrofluorescein diacetate) staining for reactive oxygen species (ROS). Epifluorescence microscope (460 nm UV filter). (**E**–**H**): Blue images Cell Tracker Blue^®^ (Invitrogen, Eugene, OR, USA) staining for glutathione (GSH). Epifluorescence microscope (405 nm UV filter). Scale bar 100 µM.

**Figure 3:**
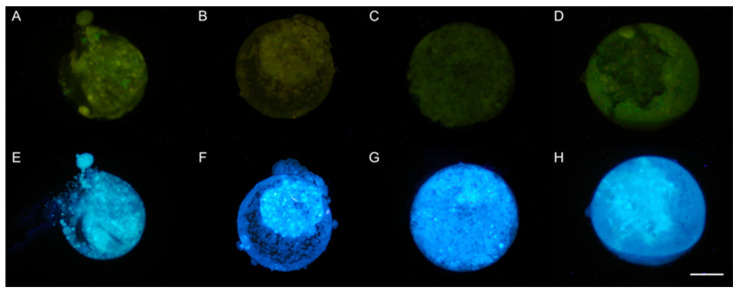
Qualitative evaluation of the presence of reactive oxygen species (ROS) and glutathione (GSH) in late blastocysts produced by in vitro fertilization (IVF) from *Ovis aries* oocytes treated with resveratrol during in vitro maturation (IVM), after devitrification. (**A**): 0 µM; (**B**): 0.25 µM; (**C**): 0.5 µM; and (**D**): 1 µM resveratrol. (**A**–**D**): Green images DCFH-DA (2′,7′-dichlorodihydrofluorescein diacetate) staining for ROS. Epifluorescence microscope (460 nm UV filter). (**E**–**H**): Blue images Cell Tracker Blue^®^ (Invitrogen, Eugene, OR, USA) staining for GSH. Epifluorescence microscope (405 nm UV filter). Scale bar 100 µM.

Before vitrification, the qualitative evaluation of the presence of reactive oxygen species (ROS) and glutathione (GSH) in blastocysts showed lower levels for ERO at 0.5 and 1 µM of resveratrol, but no differences for GSH levels between groups (Figure 2). After devitrification, the qualitative evaluation showed no differences for ERO or GSH levels in blastocysts between groups (Figure 3).

**Figure 4:**
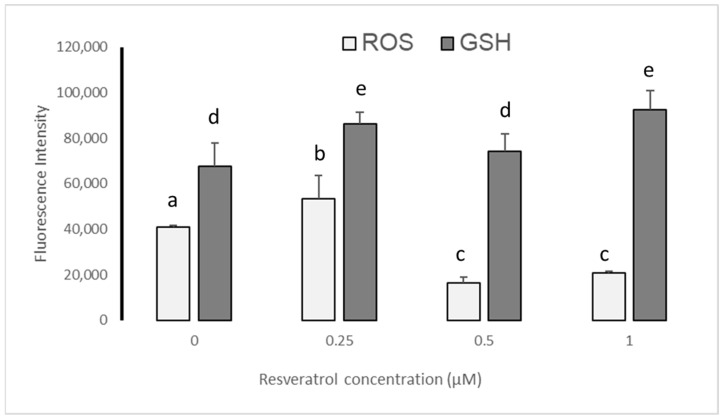
Fluorescence levels (Image J image processor version 1.53; Wayne Rasband, National Institute of Health, Kensington, MD, USA) of reactive oxygen species (ROS) and glutathione (GSH) in sheep blastocysts produced by in vitro fertilization (IVF) from *Ovis aries* oocytes treated with resveratrol, before vitrification. Literals between bars show significant differences (*p* ≤ 0.05).

Before vitrification the semiquantitative analysis given by the Image J image processor (version 1.53; Wayne Rasband, National Institute of Health, Kensington, MD, USA) determined that resveratrol reduced ROS levels only at 0.5 and 1 µM with respect to 0 and 0.25 µM (*p* ≤ 0.05); while GSH levels increased, being higher at 0.25 and 1 µM (*p* ≤ 0.05) (Figure 4).

**Figure 5:**
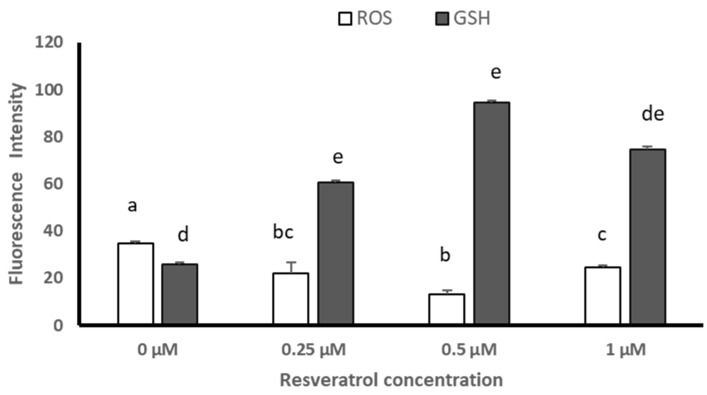
Fluorescence levels (Image J image processor version 1.53; Wayne Rasband, National Institute of Health, Kensington, MD, USA) of reactive oxygen species (ROS) and glutathione (GSH) in sheep blastocysts produced by in vitro fertilization (IVF) from *Ovis aries* oocytes treated with resveratrol, after devitrification. Literals between bars show significant differences (*p* ≤ 0.05).

After devitrification, the semiquantitative analysis given by the Image J image processor (version 1.53; Wayne Rasband, National Institute of Health, Kensington, MD, USA) determined that ROS levels were reduced with the different treatments (0.25, 0.5, and 1 µM), particularly with 0.5 µM (*p* ≤ 0.05) of resveratrol, while GSH levels increased at 0.25 and 0.5 µM of resveratrol with respect to the control group, being greater at 0.5 µM (*p* ≤ 0.05) (Figure 5).

### 3.5. DAPI Staining of Nuclei

#### 3.5.1. Presence of Nuclei in Early Blastocysts Prior to Vitrification

**Figure 6:**
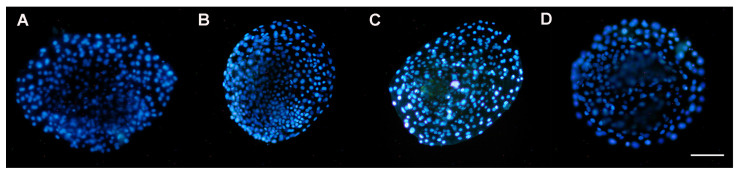
Presence of nuclei in early blastocysts prior to vitrification, produced by in vitro fertilization (IVF) from *Ovis aries* oocytes treated with resveratrol during in vitro maturation (IVM). Images (**A**): 0 µM; (**B**): 0.25 µM; (**C**): 0.5 µM; and (**D**): 1 µM resveratrol. DAPI stain. Epifluorescence microscope (405 nm UV filter). Scale bar 100 µM.

Prior to vitrification, blastocysts showed significant differences in the nuclei present between treatments, particularly for 0.5 and 1 µM of resveratrol, with respect to the control group (*p* ≤ 0.05). Significant differences were also observed between 0.25 vs. 0.5 µM and between 0.5 vs. 1 µM (*p* ≤ 0.05), with 1 µM being the treatment in which the embryos showed a greater number of nuclei, but not for 0.5 µM of resveratrol (Figure 6 and Table 3).

#### 3.5.2. Presence of Nuclei in Late Blastocysts after Devitrification

At 48 h after devitrification, the quantification of nuclei in the blastocysts showed significant differences between 0.25, 0.5, and 1 µM of resveratrol (*p* ≤ 0.05), with the number of nuclei again being greater at 1 µM. However, in all treated groups the number of nuclei present in devitrified embryos was lower than that in non-vitrified embryos (Table 4).

Although the statistical analysis revealed that treatment with 0.25 µM of resveratrol was like that of the control group before and after vitrification, 0.25 µM did not reduce the concentration of ROS in embryos prior to vitrification (Figure 4). This could have damaged the embryos’ nuclei.

## 4. Discussion

### 4.1. Effect of Resveratrol on IVM, DIV, and Blastocyst Production

It is known that the maturation rate influences the success of IVF and subsequent embryonic development. Other factors that influence this are culture media, changes in pH, osmolarity, temperature, exposure to light, concentrations of CO_2_ and oxygen, humidity, manipulation of cells, and cryopreservation methods [20,21]. If the conditions are not adequate, they will generate stress to the oocyte by affecting the cytoplasm and cell membrane, causing changes in nucleic acids, lipids, and proteins [22,23].

This oxidative stress is caused by reactive oxygen species (ROS) produced by cellular metabolism that can damage and/or kill oocytes and embryos through a decrease in ATP, blockage in embryonic development, alterations in methylation of DNA, and histone modification [20,24,25].

Reactive oxygen species are produced naturally during cellular metabolism. However, during embryonic development, especially in the transition from morula to early blastocyst, an increase in ROS occurs, and, consequently, at this stage, the blastocyst is very susceptible to oxidative stress, especially when endogenous antioxidants (enzymatic and non-enzymatic) do not achieve balance. This is why there is a need to add antioxidants to the culture media [4]. As the embryo progresses in development, cellular metabolism increases, and cells are more susceptible to oxidative stress, specifically in the mitochondrial respiratory chain. Complexes I and II produce superoxide and nitrile radicals that affect mitochondrial proteins and the function of different metabolic enzymes in the electron transport chain [26]. It has been reported that mitochondrial DNA (mtDNA) is more sensitive to oxidative stress than nuclear DNA [27], possibly because mtDNA lacks histones, which protect against such damage. Furthermore, it does not have an efficient repair system and is located near the inner mitochondrial membrane, the site of greatest ROS production [28,29]. Additionally, oxidative damage to mtDNA can induce mutations and alter mitochondrial function and integrity [30]. This oxidation–reduction imbalance in humans induces degenerative mitochondrial diseases such as Alzheimer’s, Parkinson’s, and amyotrophic lateral sclerosis [31,32].

In an in vivo system, cells are protected from these effects by the enzymatic and non-enzymatic antioxidants present; for example, oocytes are protected by ovarian follicle fluid [33]. In contrast, in in vitro systems, antioxidants are added [34], such as α-tocopherol, vitamins, proteins, antioxidant enzymes, thiolic compounds, metal chelators, vitamin E and its derivatives, cysteamine, ascorbic acid, and extracts of plants with antioxidant properties [34,35]. At physiological concentrations, ROS are essential for cell metabolism and signaling, while their overproduction will generate alterations [36,37].

Resveratrol is an antioxidant used in in vitro oocyte culture media because it promotes higher IVM rates. At 0.25 and 0.5 µM, resveratrol increases cleavage and blastocyst rates [4,7]. In the present study, oocytes treated with 0.5 and 1 μM resveratrol improved IVM, embryonic cleavage rate, and blastocyst production. It is known that the addition of this antioxidant during IVM favors the cytoplasmic and nuclear maturation of the oocyte. This effect could be related to its ability to increase the expression and nuclear translocation of transcription factors that prevent oxidative stress in the oocyte [3]. It could also be because it increases the concentration of intracellular reduced glutathione (GSH) and favors the concentrations of the enzyme glutathione peroxidase, essential for the embryonic oxidant balance [4]. This allows a decrease in ROS production, as has been observed in pigs, cattle, sheep, and goats [2,3,5,6,7,8,9,10,11].

### 4.2. Effect of Resveratrol on Embryo Vitrification and Devitrification

Vitrification subjects the cell to significant thermal and oxidative stress. In the present study, ethylene glycol was used as a cryoprotectant since its efficiency in the vitrification of ovine and bovine embryos has been reported [16,38]. It has properties such as high penetration speed and low toxicity, in addition to avoiding removing the cryoprotectant prior to embryo transfer; that is, it does not interfere with embryo implantation in embryo recipient females [39].

On the other hand, the open pulled straw (OPS) system allowed embryonic survival after vitrification (64%) at the highest concentration of resveratrol (1 µM), although it was lower than that reported by González [18], who obtained data greater than 70%.

Embryos are exposed to oxidative stress during vitrification, affecting rates of IVM, IVD, and early blastocysts when compared to in vivo systems [4,5]. However, in the present study, resveratrol supplemented during IVM reduced ROS levels in blastocysts after vitrification. Giaretta et al. [40] and Salzano et al. [41] reported that this antioxidant, when added to the culture medium, has a positive effect on development and cryotolerance of blastocysts.

### 4.3. Effect of Resveratrol on ROS and GSH Levels in Devitrified Embryos

In the present study, the vitrification and devitrification methods to which sheep embryos produced by IVF were subjected allowed a reduction in ROS levels, which was minimal at 0.5 µM, and an increase in GSH levels, reaching the highest levels at this concentration. Greater embryonic survival was achieved after devitrification at 0.5 µM and 1 µM of resveratrol (*p* < 0.05). This demonstrates that resveratrol, used in the IVM of oocytes, reduces embryonic oxidative stress by promoting the synthesis of endogenous antioxidants such as GSH, as Kwak et al. [7] report that 0.5 and 2.0 μm of resveratrol showed a significant (*p* < 0.05) increase in intracellular GSH levels in mature oocytes compared with the control group.

Glutathione is the main non-protein sulfide component in mammalian cells and is recognized for protecting the cell from oxidative damage and, regulating the intracellular redox balance [42]. It may also be important in biological processes such as DNA and protein synthesis and cell proliferation during embryonic development [43]. In cattle, it has been considered an important marker of oocyte viability and quality [44]. Additionally, GSH synthesis during in vitro maturation is associated with the formation of the male pronucleus after fertilization [45,46] and early embryonic development [43].

During embryonic development, GSH is important in DNA and protein synthesis and cell proliferation [44]. In cattle, it has been considered an important biochemical marker of oocyte viability and quality [43,46].

### 4.4. Effect of Resveratrol on Embryo Quality

Hernández et al. [47] reported a low number of nuclei (44.2 ± 9.9) present in parthenogenetic blastocysts of *O. aries* and in interspecies cloned blastocysts of *O. aries* and *Ovis canadensis* mexicana (46.7 ± 8.1 nuclei). Lorenzo-Torres et al. [48] reported 122 nuclei in good-quality blastocysts produced by IVF. These results are lower than those obtained in the present study, but they are similar to those reported by Agata et al. [49], Mastrorocco et al. [50], and Nadri et al. [51]. In the present study, before vitrification, treatments with 0.25 and 0.5 µM of resveratrol were statistically similar between them, although 0.5 µM showed the lowest number of nuclei, with 1 µM being the treatment that gave the highest number of nuclei in early blastocysts. After devitrification, although the number of nuclei decreased, 0.5 and 1 µM of resveratrol were similar between them, with the highest value also at 1 µM of resveratrol for the number of nuclei in late blastocysts.

In both cases, 1 µM of resveratrol overcame the number of nuclei of the control group and the other treatments.

It is shown that resveratrol not only favored the IVM rate of sheep oocytes but also had a positive effect on the production of higher-quality embryos and conferred cryotolerance capacity during the vitrification process. Further studies are needed to know how GSH and ERO levels behave in intact oocytes in this species.

## 5. Conclusions

It is concluded that the use of resveratrol in sheep oocytes during in vitro maturation has a beneficial effect on embryos produced by IVF subjected to vitrification by decreasing reactive oxygen species levels and facilitating the generation of the embryos’ antioxidant enzymes like glutathione.

## 6. Patents

Part of the methodology applied in this study was based on the patent No. 394003.

## Figures and Tables

**Table 1:** Rate of in vitro maturation (IVM), in vitro development (IVD), and *Ovis aries* blastocysts from oocytes treated with resveratrol.

Resveratrol	0 µM	0.25 µM	0.5 µM	1 µM
IVM	75 ± 6.5 ^a^	74 ± 8.5 ^a^	81 ± 6.1 ^b^	81 ± 6.5 ^b^
Cleavage (IVD)	63.1 ± 5.4 ^a^	60 ± 4.6 ^a^	68.6 ± 4.7 ^b^	69.2 ± 4.7 ^b^
Early Blastocysts	8.4 ± 3.4 ^a^	13.5 ± 4.1 ^a^	16.2 ± 6 ^a^	16.8 ± 6.5 ^a^
Late Blastocysts	30.2 ± 3.9 ^a^	25.6 ± 3.1 ^a^	30.2 ± 3.5 ^a^	31.4 ± 4.5 ^a^

Different literals a–b show significant differences in rows at *p* ≤ 0.05 (mean ± SE). Considering an *n* in IVM = 2204 oocytes, IVD *n* = 2204 oocytes and embryos, and blastocysts *n* = 1100 embryos.

**Table 2:** Rate of late blastocysts produced by in vitro fertilization (IVF) from *Ovis aries* oocytes treated with resveratrol during in vitro maturation (IVM), after devitrification.

Resveratrol				
Late Blastocysts	0 µM	0.25 µM	0.5 µM	1 µM
Mean ± SE	58 ± 20.9 ^a^	41 ± 23.6 ^a^	59 ± 7.3 ^a^	64 ± 13.8 ^a^

Same literal in a row shows no statistical differences (*p* ≥ 0.05).

**Table 3:** Number of nuclei present in early blastocysts prior to vitrification, produced by in vitro fertilization (IVF) from *Ovis aries* oocytes treated with resveratrol during in vitro maturation (IVM).

Variable	Resveratrol Concentration			
Number of nuclei	0 µM	0.25 µM	0.5 µM	1 µM
Mean ± SE	267 ± 28.8 ^a^	260 ± 33.2 ^ac^	185 ± 11.7 ^bc^	354 ± 21.5 ^d^

Literals a-b-c-d: between lines show significant differences between treatments (*p* ≤ 0.05). Day 7 of culture.

**Table 4:** Number of nuclei present in blastocysts produced by in vitro fertilization (IVF) from *Ovis aries* oocytes treated with resveratrol during in vitro maturation IVM, after devitrification.

Variable	Resveratrol Concentration			
Number of nuclei	0 µM	0.25 µM	0.5 µM	1 µM
Mean ± SD	109 ± 4.3 ^a^	90 ± 12.3 ^a^	147 ± 21 ^b^	150 ± 19.4 ^b^

Literals a-b: between rows show significant differences between treatments (*p* ≤ 0.05). Forty-eight hours after devitrification.

## Data Availability

Data are available from the first author Andrea Carolina González Garzón (andrea.gonzalez.garzon@unillanos.edu) upon request.
